# Association of Individual and Community Factors With Hepatitis C Infections Among Pregnant People and Newborns

**DOI:** 10.1001/jamahealthforum.2021.3470

**Published:** 2021-10-29

**Authors:** Stephen W. Patrick, William D. Dupont, Elizabeth McNeer, Melissa McPheeters, William O. Cooper, David M. Aronoff, Sarah Osmundson, Bradley D. Stein

**Affiliations:** 1 Vanderbilt Center for Child Health Policy, Vanderbilt University Medical Center, Nashville, Tennessee; 2 Department of Pediatrics, Vanderbilt University Medical Center, Nashville, Tennessee; 3 Department of Health Policy, Vanderbilt University Medical Center, Nashville, Tennessee; 4 RAND Corporation, Pittsburgh, Pennsylvania; 5 Department of Biostatistics, Vanderbilt University Medical Center, Nashville, Tennessee; 6 Department of Medicine, Division of Infectious Disease, Vanderbilt University Medical Center, Nashville, Tennessee; 7 Department of Obstetrics and Gynecology, Vanderbilt University Medical Center, Nashville, Tennessee; 8 University of Pittsburgh School of Medicine, Pittsburgh, Pennsylvania

## Abstract

**Importance:**

The opioid crisis has increasingly affected pregnant people and infants. Hepatitis C virus (HCV) infections, a known complication of opioid use, grew in parallel with opioid-related complications; however, the literature informing individual and community risks associated with maternal HCV infection is sparse.

**Objectives:**

To determine (1) individual and county-level factors associated with HCV among pregnant people and their newborn infants, and (2) how county-level factors influence individual risk among the highest risk individuals.

**Design, Setting, and Participants:**

This time-series analysis of retrospective, repeated cross-sectional data included pregnant people in all US counties from 2009 to 2019. We constructed mixed-effects logistic regression models to explore the association between HCV infection and individual and county-level covariates. Analyses were conducted between June 2019 and September 2021.

**Exposures:**

Individual-level: race and ethnicity, education, marital status, insurance type; county-level: rurality, employment, density of obstetricians.

**Main Outcomes and Measures:**

Hepatitis C virus as indicated on the newborn’s birth certificate.

**Results:**

Between 2009 and 2019, there were 39 380 122 pregnant people who met inclusion criteria, among whom 138 343 (0.4%) were diagnosed with HCV. People with HCV were more likely to be White (79.9% vs 53.5%), American Indian or Alaska Native (AI/AN) (2.9% vs 0.9%), be without a 4-year degree (93.2% vs 68.6%), and be unmarried (73.7% vs 38.8%). The rate (per 1000 live births) of HCV among pregnant people increased from 1.8 to 5.1. In adjusted analyses, the following factors were associated with higher rates of HCV: individuals identified as White (adjusted odds ratio [aOR], 7.37; 95% CI, 7.20-7.55) and AI/AN (aOR, 7.94; 95% CI, 7.58-8.31) compared with Black individuals, those without a 4-year degree (aOR, 3.19; 95% CI, 3.11-3.28), those with Medicaid vs private insurance (aOR, 3.27; 95% CI, 3.21-3.33), and those who were unmarried (aOR, 2.80; 95% CI, 2.76-2.84); whereas, rural residence, higher rates of employment, and greater density of obstetricians was associated with lower risk of HCV. Among individuals at the highest risk of HCV, higher levels of county employment, accounting for other factors, were associated with less of a rise in HCV infections over time.

**Conclusions and Relevance:**

In this cross-sectional study, maternal and newborn HCV infections increased substantially between 2009 and 2019, disproportionately among White and AI/AN people without a 4-year degree. County-level factors, including higher levels of employment, were associated with lower individual risks of acquiring the virus.

## Key Points

**Question:**

What individual and county factors are associated with hepatitis C virus (HCV) infection among pregnant people and which county factors modify risk among those at highest risk?

**Findings:**

In this retrospective repeated cross-sectional study of US counties and 39 380 122 pregnant people with live births, White and American Indian/Alaska Native people without a 4-year college degree had the highest individual risk of HCV. High levels of county employment mitigated the rise of HCV infections among people with the highest risk of acquiring the virus.

**Meaning:**

US HCV infections among pregnant people grew fastest among White and American Indian/Alaska Native people without a 4-year degree; however, county-level factors, including higher levels of employment, modified this risk.

## Teaser

This cross-sectional study examines individual and community factors associated with hepatitis C virus risk among pregnant people and their newborn infants.

## Introduction

The opioid crisis has taken a substantial toll on pregnant people and infants in the US. Over the last 20 years, rates of opioid use disorder (OUD) among pregnant people^1,2^ and neonatal opioid withdrawal syndrome (NOWS)^2,3,4^ among infants grew exponentially. In parallel, there is increasing recognition that hepatitis C virus (HCV) infections, the most common bloodborne infection in the US, are a growing public health problem for maternal child health. Infection rates have substantially increased among pregnant people,^5^ especially among those with a diagnosis of OUD,^6^ and there has been a corresponding increase in infant exposures to the virus in many communities.^7,8^ Individuals who use opioids intravenously are at high risk of acquiring hepatitis C virus (HCV),^9^ which can also lead to vertical transmission to their infant.^10^ Perhaps because HCV has increased in communities in the US, there has been a recent rise in HCV testing of pregnant people and infants along with an increase in the positivity rate for both.^11^ Even with this recent growth, individual and community-level factors associated with HCV among pregnant people have not been well defined. Without evidence of factors associated with HCV risk for pregnant people, interventions to protect the health of both pregnant people and infants may be ineffective.

It is increasingly clear that particular social determinants of health are associated with many adverse health outcomes^12,13,14^ and with increased risk of being diagnosed with OUD and NOWS in pregnant people and infants. Across the total population, the recent rise in deaths by suicide, overdose, and alcoholic liver disease, collectively termed “deaths of despair,” occurred disproportionately among individuals with lower levels of employment and education, and greater levels of poverty, suggesting these factors may also influence opioid-related adverse pregnancy outcomes.^15^ Alternatively, the literature suggests that some social factors, including education and employment,^16^ may be protective against adverse outcomes. Although the relationship between community-level factors and NOWS, reflecting maternal opioid use proximal to pregnancy has been studied,^17^ to our knowledge the relationship of community-level factors, aside from rurality,^18^ and increased HCV-risk in pregnant people has not been evaluated. Informed by the existing literature,^15,17^ we hypothesized that access to health care (density of obstetricians) and employment would be associated with a decrease in risk of HCV among pregnant people, whereas residing in a rural county would be associated with an increased risk of acquiring the virus. With constrained national resources, understanding both individual and community-level factors associated with HCV infections in pregnant people could inform strategies to mitigate its spread, such as harm reduction efforts (eg, syringe service programs), improving access to treatment for OUD or increasing the obstetrical workforce in high-risk communities, HCV testing strategies in pregnant people and people of childbearing age, and treatment with novel antiviral therapies. To address these knowledge gaps, we examined individual and county-level factors associated with the rise of HCV among pregnant people in the US and explored interactions between them.

## Methods

This retrospective, repeated cross-sectional study included data from all US births from 2009 to 2019. Data were obtained from natality files obtained from the National Center for Health Statistics at the Centers for Disease Control and Prevention and from the Area Health Resource File (AHRF). Our approach focused on how community-level factors may be associated with changes in HCV risk among pregnant people. Opioid-related complications have disproportionately occurred among non-Hispanic White^3,4,5,19^ and American Indian/Alaska Native (AI/AN)^20^ pregnant people and infants compared with other populations and among less educated pregnant people.^5^ Informed by the existing literature,^15,17^ we examined community level factors in 3 domains: rurality, employment, and access to medical care. The Vanderbilt University Medical Center institutional review board deemed this study as nonhuman subjects research given its use of deidentified data and was therefore granted a waiver of informed consent. This study followed the Strengthening the Reporting of Observational Studies in Epidemiology (STROBE) reporting guidelines. Analyses were conducted between June 2019 and September 2021.

### Cohort and Data

The cohort included all pregnant people in the US who experienced live births between 2009 and 2019 in counties where HCV infection was recorded (>99% by 2014). Reporting of HCV on the birth certificate became standardized as states adopted the Centers for Disease Control and Prevention’s Standard Birth Certificate.

### Outcome and Covariates

Our outcome of interest was maternal HCV infection as indicated on the birth certificate of the newborn. Birth data were not included if HCV status was unknown or missing. Maternal race and ethnicity as identified on the birth certificate (AI/AN, Black, Hispanic, White, other, education [with and without 4-year degree]), number of previous births, marital status, and birth payment source (Medicaid, private insurance, self-pay, other) were also obtained from the National Vital Statistics System. Using the AHRF, we calculated the number of employed per 1000 population and the number of obstetricians per 1000 population in each county. Rurality was determined using Rural-Urban Continuum Codes and grouped into categories for urban, rural adjacent, and rural remote.^17^

### Data Analysis

Line plots were used to examine trends in maternal HCV rates per 1000 births, and descriptive statistics (Pearson χ^2^ and Wilcoxon rank-sum tests as appropriate) were calculated to compare maternal characteristics between people with HCV and those without HCV.

Our analysis was conducted in 2 phases. First, using the entire cohort we constructed a mixed-effects (random intercepts for county, fixed effects for covariates) logistic regression model to explore the association between HCV infection and individual and county-level covariates. Model covariates, chosen a priori, were year of birth, race, education (with and without 4-year degrees), number of previous births, insurance type, and marital status, rurality (urban, rural adjacent, and remote), proportion of employed population, and density of obstetricians. The rurality and density covariates were attributes of the county where the patient lived. All other covariates were attributes of the patient.

Next, to better understand how county-level factors may be associated with risks for HCV for pregnant people identified to be at the highest risk of acquiring HCV in the first phase (AI/AN and White pregnant people without a 4-year degree), we tested interaction terms consisting of year and each county-level variable (rurality, employment, density of obstetricians). Restricted cubic splines were used for nonlinear predictors. To facilitate the interpretation of the effect of county-level covariates, we plotted the predicted probability of HCV infection over time for different values of each of the county-level covariates. Lastly, we mapped HCV prevalence among pregnant people in each US county during our study period. Statistical significance was set at *P* < .05 for all tests, which were 2-sided. Statistical analyses were conducted using R (version 3.5.1, The R Foundation for Statistical Computing) and Stata statistical software (version 14.2, Stata Corp). Analyses were conducted between June 2019 and September 2021.

## Results

Between 2009 and 2019, there were a total of 39 380 122 pregnant people who had live births residing in counties reporting HCV, among whom 138 343 (0.4%) were diagnosed with HCV. The overall rate (per 1000 live births) of HCV in pregnant people increased from 1.8 to 5.1 between 2009 and 2019.

**Figure 1.**
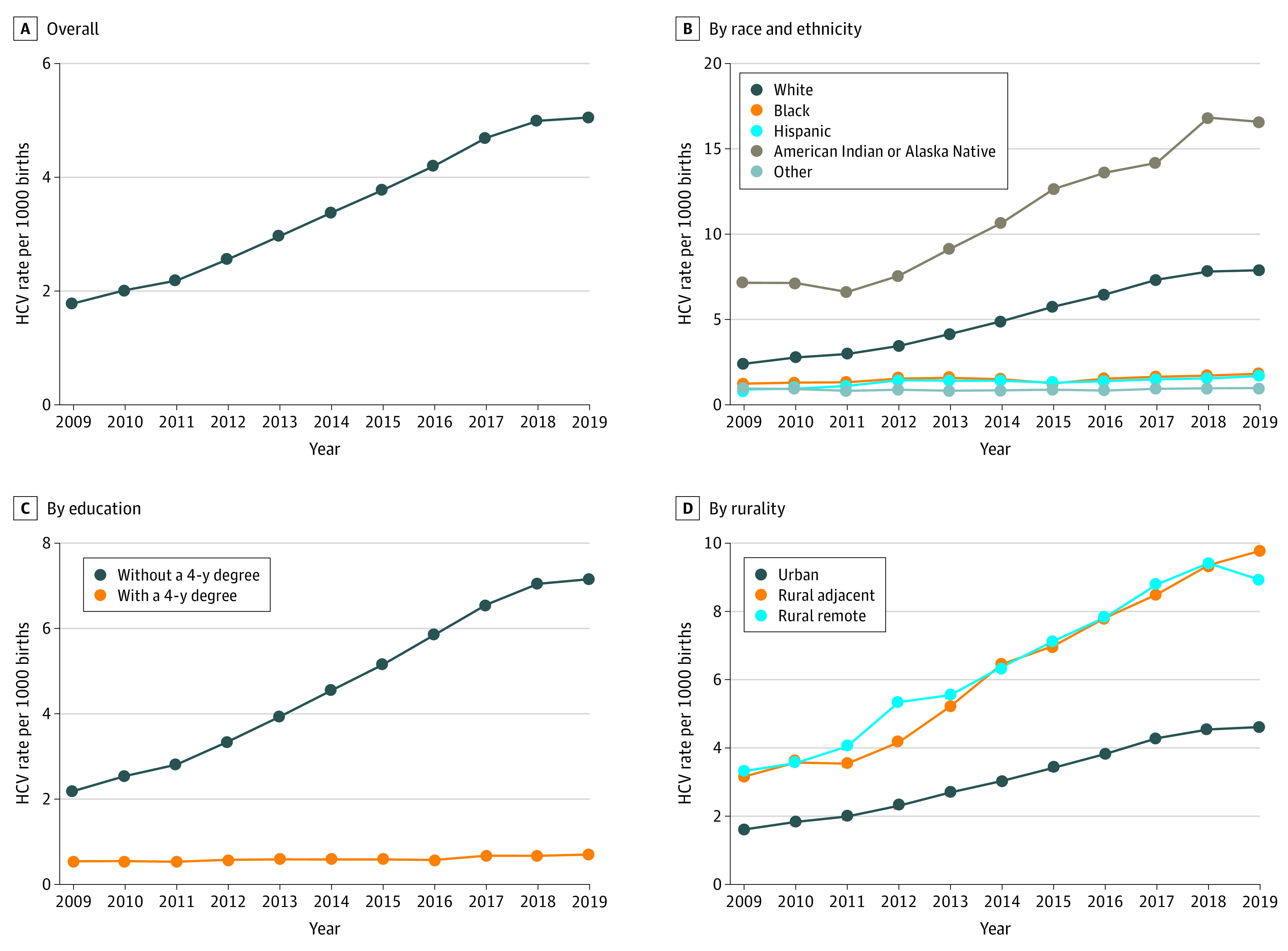
Hepatitis C Infections Among Pregnant People Delivering Live Births in the US, 2009 to 2019 A, Overall; B, stratified by race; C, education; and D, rurality.

Unadjusted data suggested that people diagnosed with HCV were more likely to be White (79.9% vs 53.5%), AI/AN (2.9% vs 0.9%), have less than a 4-year college degree (93.2% vs 68.6%), be insured by Medicaid (76.7% vs 42.6%), be unmarried (73.7% vs 38.8%), and less likely to reside in an urban county (81.8% vs 90.0%; Table 1, *P* < .001). There were substantial differences in the rate of change of the diagnosis by race and ethnicity, education, and rurality. Overall, HCV rates (per 1000 births) increased greatest among AI/AN (7.2 to 16.6) and White (2.4 to 7.9) pregnant people, compared with Black pregnant people among whom the rate was relatively unchanged (1.2 to 1.8). Rates among people without a 4-year degree increased from 2.2 to 7.2; whereas rates in people with a 4-year degree remained stable, increasing only from 0.5 to 0.7. Rates among people residing in rural counties increased (rural adjacent, 3.2-9.8; rural remote, 3.3-8.9) to higher levels than among those in urban communities (1.6 to 4.6; Figure 1). When stratified by race and ethnicity and education, pregnant people at highest risk of HCV were AI/AN and White individuals without a 4-year degree (eFigure 1 in the Supplement).

**Table 1.** Characteristics of Pregnant People in the US Delivering Live Infants With and Without Hepatitis C Infections, 2009 to 2019

Characteristic	Hepatitis C virus, No. (%)	
	Positive (n = 138 343)	Negative (n = 39 241 779)
Maternal race and ethnicity		
American Indian or Alaska Native	4044 (2.9)	349 569 (0.9)
Black	8792 (6.4)	5 814 143 (14.8)
Hispanic	12 650 (9.1)	9 446 451 (24.1)
White	110 507 (79.9)	21 010 193 (53.5)
Other^a^	2.350 (1.7)	2 621 423 (6.7)
Maternal age, median (IQR), y	28 (25-32)	28 (24-33)
Maternal education		
Without a 4-y degree	128 946 (93.2)	26 933 174 (68.6)
With a 4-y degree	7197 (5.2)	11 819 756 (30.1)
No. of previous births, median (IQR)	1 (0-2)	1 (0-2)
Insurance		
Medicaid	106 158 (76.7)	16 722 986 (42.6)
Private insurance	20 556 (14.9)	18 724 355 (47.7)
Self-pay	3945 (2.9)	1 681 108 (4.3)
Other	5805 (4.2)	1 708 194 (4.3)
Marital status		
Married	34 725 (25.1)	22 644 619 (57.7)
Unmarried	101 943 (73.7)	15 223 397 (38.8)
Rurality		
Rural		
Adjacent	14 383 (10.4)	2 293 331 (5.8)
Remote	10 767 (7.8)	1 650 463 (4.2)
Urban	113 193 (81.8)	35 297 941 (90.0)

^a^ Other was Asian or Pacific Islander.

### Multivariable Analysis

In the model developed using the entire cohort, HCV rates were highest in AI/AN (aOR, 7.94; 95% CI, 7.58-8.31) and White people (aOR, 7.37; 95% CI, 7.20-7.55) compared with Black pregnant people. In addition, individuals without a 4-year degree were at higher risk of HCV (aOR, 3.19; 95% CI, 3.11-3.28) compared with those with a 4-year degree, unmarried people (aOR, 2.80; 95% CI, 2.76-2.84) compared with married people, and Medicaid (aOR, 3.27; 95% CI, 3.21-3.33) compared with private insurance. Residing in a rural-adjacent (aOR 0.80; 95% CI, 0.75-0.87) or rural-remote (aOR 0.80, 95% 0.73-0.88) compared with an urban area, proportion of county employed (aOR, 1.00; 95% CI, 1.00-1.00), and density of obstetricians (aOR, 0.71; 95% CI, 0.51-0.99) were associated with lower rates of HCV infections among pregnant people (Table 2).

**Table 2.** Multivariable Analysis of Individual and County-Level Factors Associated With Hepatitis C Virus Among Pregnant People Having Live Births in the US, 2009 to 2019

Variable	aOR (95% CI)
Year	1.13 (1.12-1.13)^a^
Individual characteristics	
Race and ethnicity	
American Indian or Alaska Native	7.94 (7.58-8.31)^a^
Black	1 [Reference]
Hispanic	1.45 (1.41-1.50)^a^
White	7.37 (7.20-7.55)^a^
Other	2.86 (2.72-3.00)^a^
Education	
With a 4-y degree	1 [Reference]
Without a 4-y degree	3.19 (3.11-3.28)^a^
Previous births	1.28 (1.28-1.29)^a^
Payment	
Private insurance	1 [Reference]
Self-pay	1.92 (1.85-1.99)^a^
Other	2.89 (2.80-2.98)^a^
Medicaid	3.27 (3.21-3.33)^a^
Marital status	
Married	1 [Reference]
Unmarried	2.80 (2.76-2.84)^a^
County characteristics	
Rurality	
Urban	1 [Reference]
Rural	
Adjacent	0.80 (0.75-0.87)^a^
Remote	0.80 (0.73-0.88)^a^
Employed per 1000	1.00 (1.00-1.00)^a^
Obstetricians per 1000	0.71 (0.51-0.99)

Abbreviation: aOR, adjusted odds ratio.

^a^ *P* < .001.

### County-Level Factors Associated With HCV Risk

**Figure 2.**
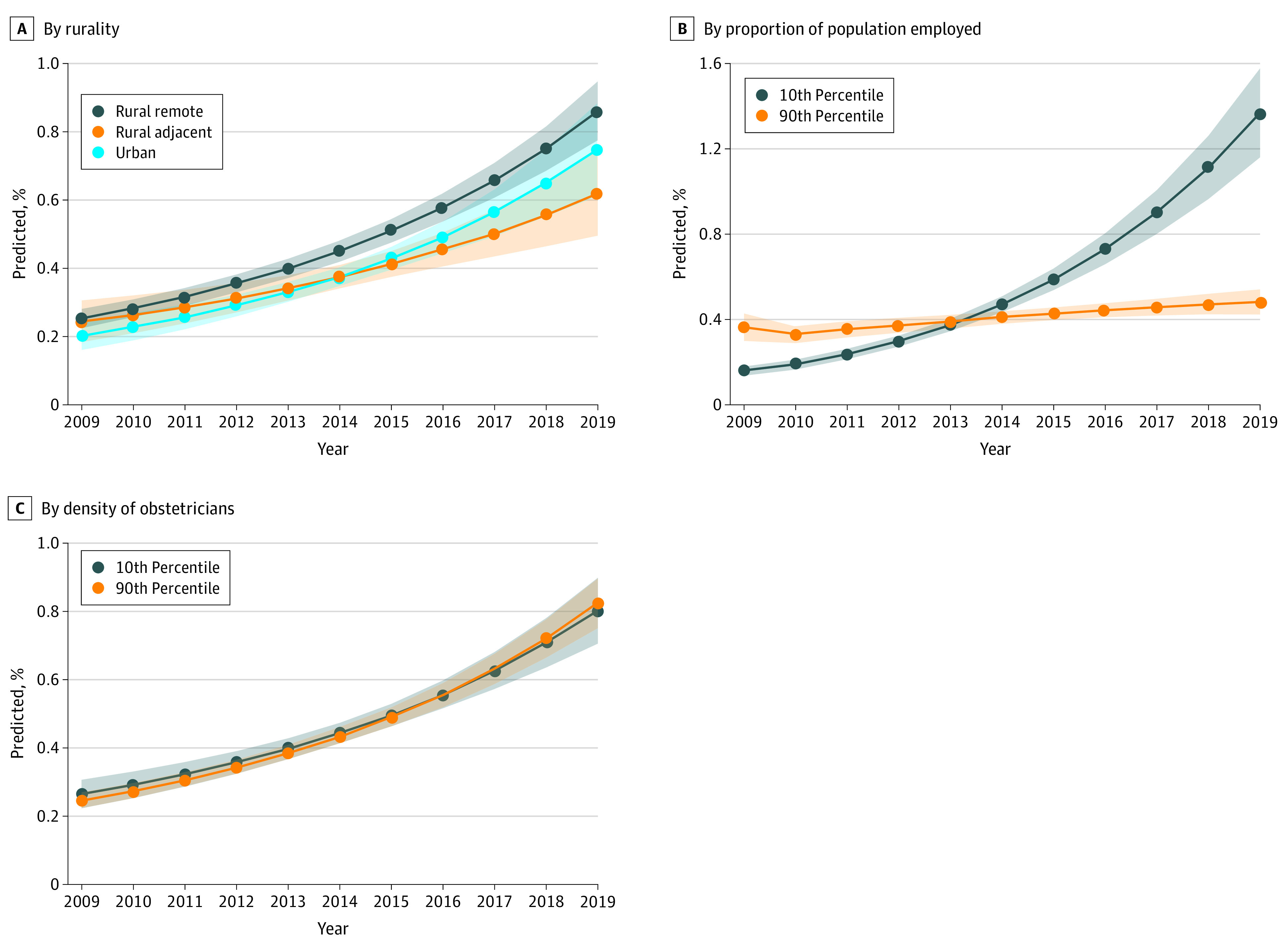
Predicted Probability of Hepatitis C Infection Among White People Without a 4-Year Degree by County Characteristics, 2009 to 2019 A, Rurality; B, proportion of population employed; and C, density of obstetricians.

To understand how county-level factors may modify HCV risk among pregnant people with the highest risk of HCV (AI/AN and White people without a 4-year degree), we added the interactions of county-level factors (rurality, employment, density of obstetricians) and year of birth to our model and then calculated predicted probabilities. We found a significant association of employment with the population with greatest risk of HCV, with greater employment at the county level associated with lower HCV risk (Figure 2; eTable 1 in the Supplement). For counties in the 10th percentile of employment, the predicted probability of HCV among high-risk pregnant people increased from 0.16% (95% CI, 0.14%-0.18%) in 2009 to 1.37% (95% CI, 1.16%-1.58%) in 2019. In contrast, risk of HCV for that same population in counties at the 90th percentile of employment remained unchanged during this time, with a predicted probability of 0.36% (95% CI, 0.30%-0.43%) in 2009 and 0.48% (95% CI, 0.42%-0.54%) in 2019. Rurality and availability of an obstetrician were not associated with changes in HCV risk (eTable 2 in the Supplement).

### Geographic Variation in HCV

**Figure 3.**
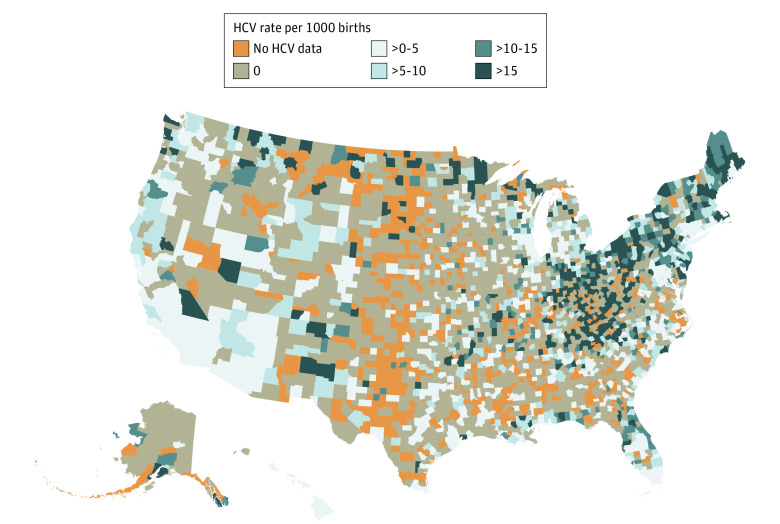
County Geographic Variation in Hepatitis C Infections Among Pregnant People in the US, 2019

In 2019, about half (49.6%) of counties in the US had at least 1 case of HCV, and most births (96.0%) occurred in those counties. The median (IQR) HCV rate in those counties was 6.36 (2.90-14.45) per 1000 births. Counties with the highest rates tended to be in the Northeast and in Appalachia. For example, among counties with at least 100 births, 8 of the top 10 counties of maternal HCV prevalence in the US were in Appalachia, including 4 in Kentucky, 1 in North Carolina, 1 in Tennessee, 1 in Virginia, 1 in West Virginia, 1 in Montana, and 1 in North Dakota. A total of 1197 of 2571 counties (46.6%) reached the prevalence threshold of 0.1% or greater and these counties accounted for 2 850 479 total births (76%; Figure 3) of the 3 747 882 US births during 2019. Geographic variation from 2009 and 2018 can be found in eFigures 2 to 11 in the Supplement.

## Discussion

Between 2009 and 2019, HCV infections more than doubled among pregnant people in the US; however, this increase varied substantially by individual characteristics and county factors. We found that AI/AN and White people without a 4-year college degree were at highest risk of having HCV. Furthermore, county-level factors were also associated with different levels of risk, with the proportion of population employed associated with lower levels of risk of HCV over time. Although individual and county-level factors associated with HCV among pregnant people have been poorly understood, they appear to mirror many of the dynamics associated with the opioid crisis.^2,15,17^

In the present study, AI/AN and White race, particularly when combined with lower education, were associated with higher HCV diagnoses, regardless of community measures. This resembles research on deaths of despair described by economists Case and Deaton as deaths due to suicide, alcohol, and overdose.^15^ Similar to the findings of their work, we found that AI/AN and White pregnant people without a 4-year degree were the most likely group to be diagnosed with HCV. However, even the group of people at highest risk based on these individual factors experienced different probabilities of HCV in counties with and without high employment, with much greater risk in those counties with low (10th percentile) employment levels. Research suggests that rural White^2^ and AI/AN people^20^ have high rates of opioid-related diagnoses during pregnancy and NOWS, and these same groups had the highest incidence of HCV in this study. However, in adjusted models, rurality was associated with decreased risk, suggesting that other factors (eg, education, employment) may account for the differential HCV risk observed in HCV prevalence between rural and urban settings. Notably, although research has consistently focused on opioid-related complications among White people,^2^ nonwhite people, and infants receive less evidence-based care for both HCV and opioid use disorder and face disproportionate challenges in the child welfare system. For example, Black pregnant people with OUD are less likely to be prescribed medications for opioid use disorder,^21^ Black infants exposed to HCV are less likely to be tested for HCV,^7^ and Black parents whose infants are placed in foster care for substance exposure are less likely to be reunified with their parents.^22,23^ As systems are developed to address the rising numbers of maternal-infant dyads affected by HCV, it is critical that any intervention is applied equitably and addresses unequal treatment in these associated systems of care.

The rise of HCV among pregnant people has substantial implications for pregnant people and infants. Hepatitis C virus is the most common bloodborne infection in the US, infecting an estimated 2.4 million people nationwide,^24^ with the chief risk factor for acquiring the virus being injection drug use.^9,25^ Although there is no FDA-approved treatment for HCV in pregnancy, identifying HCV in pregnancy is important for providing treatment^26^ for pregnant people after delivery and for monitoring infants for seroconversion. Because maternal antibodies to HCV can persist, historical recommendations have been to follow and test exposed infants at age 18 months; however, more recent recommendations include earlier RNA testing, twice before age 6 months.^25^ Although vertical transmission of HCV is rare, occurring among an estimated 6% of exposed infants (higher with greater viral load or coinfection with HIV),^27^ systems to follow and identify infants with seroconversion are underdeveloped and data suggest most exposed infants are not tested.^7,8,28^ Although several individual risk factors have been identified that increase risk of HCV among pregnant people,^5^ many of these factors can be difficult to identify in clinical practice. Recent cost-effectiveness analyses suggest that universal screening of HCV in pregnancy is cost effective.^29^ In addition, the CDC published recommendations in 2020 that communities with a prevalence of 0.1% or greater of HCV universally test pregnant people for the virus.^25^ Following the CDC recommendations, the US Preventative Services Task Force^30^ and the American College of Obstetricians and Gynecologists (ACOG) recommended universal screening of HCV in pregnancy.^31^ In our analysis, 45% of US counties accounting for 70% of US births met the 2020 CDC threshold, providing additional support for new recommendations of nationwide universal screening.

As policymakers consider efforts to improve outcomes for pregnant people and infants affected by the opioid crisis, mitigating the rise of HCV in this population should be a public health priority. Until recently, HCV testing of pregnant people was risk based, likely missing opportunities to identify infected pregnant people and exposed infants.^7^ As clinicians implement new guidelines, which aim to universally test pregnant people for HCV, it may be important to bolster medical and public health systems to ensure adequate follow-up and connection to treatment. Furthermore, maternal-child health public health systems are often delivered through a patchwork of public programs, perhaps missing touchpoints to identify and educate families at risk for HCV in public programs (eg, Special Supplemental Nutrition Program for People, Infants, and Children, Maternal, Infant, and Early Childhood Home Visiting Program). Policymakers could consider programs that improve care coordination or care between people and their infants, treatment for opioid use disorder, and management of HCV. Finally, improving access to medications for opioid use disorder among people of reproductive age and pregnant people should remain a key public health goal because this has been demonstrated to reduce injection drug use,^32^ the chief risk factor for acquiring HCV. Despite evidence that medications for opioid use disorder improve outcomes,^33^ pregnant people are less likely to be accepted to treatment for opioid use disorder than nonpregnant people.^34^

### Limitations

Our findings should be interpreted in consideration of the limitations of our analysis. First, data obtained from birth certificates may be prone to misclassification bias with errors of omission or commission. Notably, not all states reported HCV early in the study period because they had not adopted the CDC’s Standard Birth Certificate, possibly influencing our results in those years. Second, because testing of HCV is not universal, we may detect higher rates of HCV in communities that test more frequently. Further, it is possible that people with specific characteristics were more likely to be tested than others, perhaps introducing bias into the results. Third, birth certificates may incompletely document HCV among pregnant people,^35^ and may be different than direct testing of HCV infections. Fourth, the ecological nature of our study cannot determine causation between the exposures and outcomes of interest. Finally, data obtained from birth certificates do not provide detail to know the timing of the infection or its chronicity.

## Conclusions

In this cross-sectional study, HCV infections were a rising threat to maternal-child health in the US. We found that the rise of HCV occurred disproportionately among AI/AN and White pregnant people without a 4-year college degree, however, the level of risk in this population was lower in counties with higher levels of employment. Nationwide, despite rising HCV prevalence the virus has not historically been universally screened in pregnancy and public health systems to ensure maternal-infant dyads are evaluated and treated for HCV are lacking. As systems are developed to prevent, evaluate, and treat dyads at risk for HCV they should consider both the individual and community risks that may influence risk of acquiring the virus.

## Notes

Supplement. **eFigure 1.** Rate of HCV Among Pregnant People By Race and Education-Level, US 2009-2019 **eTable 1.** Multivariable Analysis of County Factors Associated with Modifying HCV Risk Over Time among High-Risk Pregnant People **eTable 2.** Predicted Probability of Hepatitis C Infection among Non-Hispanic White and American Indian or Alaska Native Women without a 4-Year Degree by County Characteristics, 2009-2019 **eFigure 2.** County Geographic Variation in Hepatitis C Infections Among Pregnant People, US 2009 **eFigure 3.** County Geographic Variation in Hepatitis C Infections Among Pregnant People, US 2010 **eFigure 4.** County Geographic Variation in Hepatitis C Infections Among Pregnant People, US 2011 **eFigure 5.** County Geographic Variation in Hepatitis C Infections Among Pregnant People, US 2012 **eFigure 6.** County Geographic Variation in Hepatitis C Infections Among Pregnant People, US 2013 **eFigure 7.** County Geographic Variation in Hepatitis C Infections Among Pregnant People, US 2014 **eFigure 8.** County Geographic Variation in Hepatitis C Infections Among Pregnant People, US 2015 **eFigure 9.** County Geographic Variation in Hepatitis C Infections Among Pregnant People, US 2016 **eFigure 10.** County Geographic Variation in Hepatitis C Infections Among Pregnant People, US 2017 **eFigure 11.** County Geographic Variation in Hepatitis C Infections Among Pregnant People, US 2018
