# Errors in Figure 2 Caption and Panel Labels

**DOI:** 10.1001/jamahealthforum.2021.4347

**Published:** 2021-12-10

In the article titled “Association of Individual and Community Factors With Hepatitis C Infections Among Pregnant People and Newborns,”^1^ published on October 29, 2021, there were errors in the caption and panel labels for Figure 2. This article was corrected online.
