# Supplementary material for: Association of Individual and Community Factors With Hepatitis C Infections Among Pregnant People and Newborns
Source: JAMA Health Forum. 2021 Oct 29;2(10):e213470. doi: 10.1001/jamahealthforum.2021.3470 (PMC8727040; doi:10.1001/jamahealthforum.2021.3470)
Supplement: Supplement. — eFigure 1. Rate of HCV Among Pregnant People By Race and Education-Level, US 2009-2019 eTable 1. Multivariable Analysis of County Factors Associated with Modifying HCV Risk Over Time among High-Risk Pregnant People eTable 2. Predicted Probability of Hepatitis C Infection among Non-Hispanic White and American Indian or Alaska Native Women without a 4-Year Degree by County Characteristics, 2009-2019 eFigure 2. County Geographic Variation in Hepatitis C Infections Among Pregnant People, US 2009 eFigure 3. County Geographic Variation in Hepatitis C Infections Among Pregnant People, US 2010 eFigure 4. County Geographic Variation in Hepatitis C Infections Among Pregnant People, US 2011 eFigure 5. County Geographic Variation in Hepatitis C Infections Among Pregnant People, US 2012 eFigure 6. County Geographic Variation in Hepatitis C Infections Among Pregnant People, US 2013 eFigure 7. County Geographic Variation in Hepatitis C Infections Among Pregnant People, US 2014 eFigure 8. County Geographic Variation in Hepatitis C Infections Among Pregnant People, US 2015 eFigure 9. County Geographic Variation in Hepatitis C Infections Among Pregnant People, US 2016 eFigure 10. County Geographic Variation in Hepatitis C Infections Among Pregnant People, US 2017 eFigure 11. County Geographic Variation in Hepatitis C Infections Among Pregnant People, US 2018 [file jamahealthforum-e213470-s001.pdf]

Supplemental Online Content

Patrick SW, Dupont WD, McNeer E, et al. Association of Individual and Community Factors With Hepatitis C Infections Among Pregnant People and Newborns. *JAMA Health Forum*. 2021;2(10):e213470. doi:10.1001/jamahealthforum.2021.3470

eFigure 1. Rate of HCV Among Pregnant People By Race and Education-Level, US 2009-2019
eTable 1. Multivariable Analysis of County Factors Associated with Modifying HCV Risk Over Time among High-Risk Pregnant People
eTable 2. Predicted Probability of Hepatitis C Infection among Non-Hispanic White and American Indian or Alaskan Native Women without a 4-Year Degree by County Characteristics, 2009-2019
eFigure 2. County Geographic Variation in Hepatitis C Infections Among Pregnant People, US 2009
eFigure 3. County Geographic Variation in Hepatitis C Infections Among Pregnant People, US 2010
eFigure 4. County Geographic Variation in Hepatitis C Infections Among Pregnant People, US 2011
eFigure 5. County Geographic Variation in Hepatitis C Infections Among Pregnant People, US 2012
eFigure 6. County Geographic Variation in Hepatitis C Infections Among Pregnant People, US 2013
eFigure 7. County Geographic Variation in Hepatitis C Infections Among Pregnant People, US 2014
eFigure 8. County Geographic Variation in Hepatitis C Infections Among Pregnant People, US 2015
eFigure 9. County Geographic Variation in Hepatitis C Infections Among Pregnant People, US 2016
eFigure 10. County Geographic Variation in Hepatitis C Infections Among Pregnant People, US 2017
eFigure 11. County Geographic Variation in Hepatitis C Infections Among Pregnant People, US 2018

This supplemental material has been provided by the authors to give readers additional information about their work.

© 2021 Patrick SW et al. *JAMA Health Forum.*

eFigure 1. Rate of HCV Among Pregnant People By Race and Education-Level, US 2009-2019.

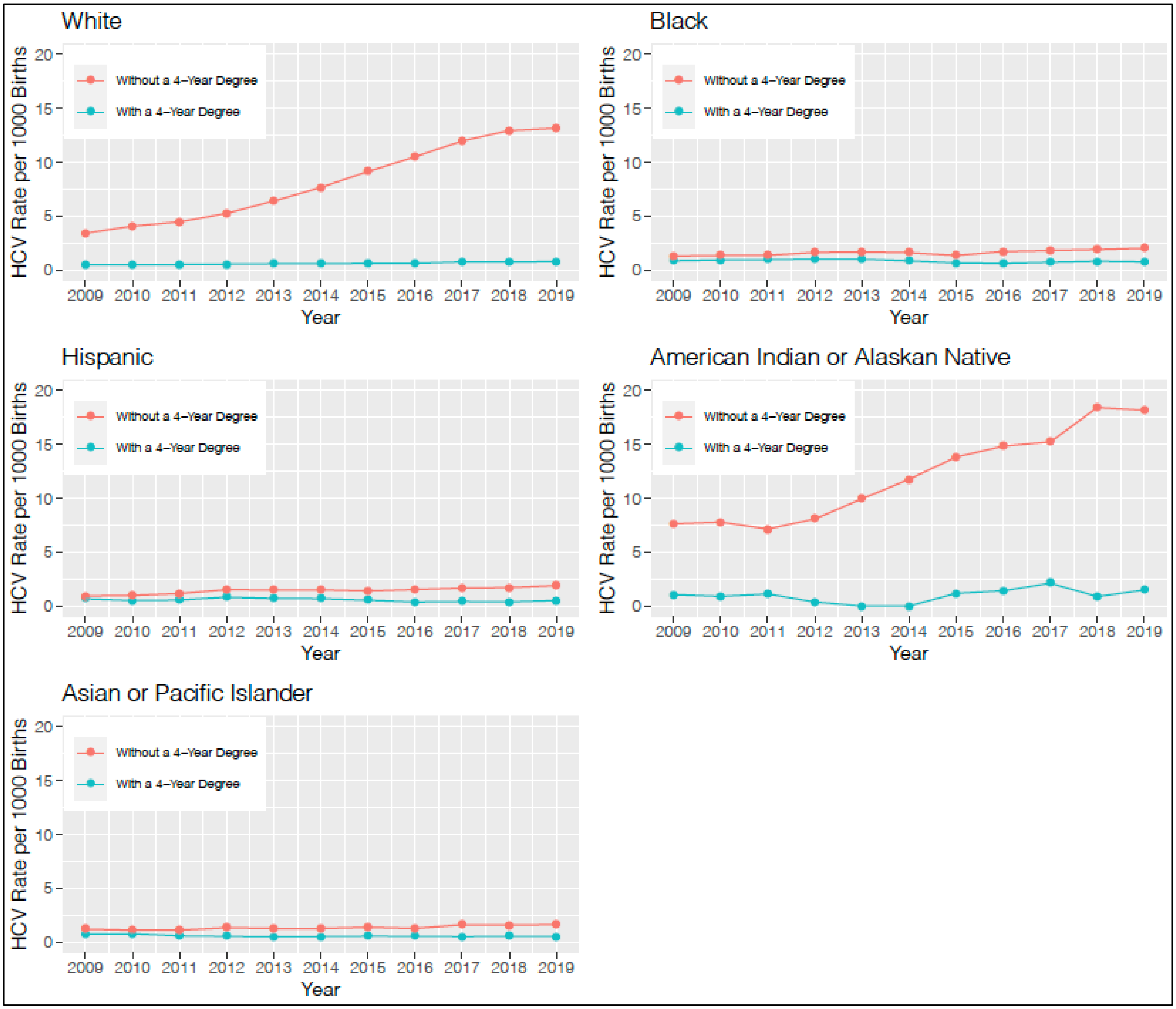

© 2021 Patrick SW et al. *JAMA Health Forum.*

eTable 1. Multivariable Analysis of County Factors Associated with Modifying HCV Risk Over Time among High-Risk Pregnant People.

	**Model Including Only White and American Indian or Alaskan Native People without a 4-year Degree** OR (95% C.I.)
**Year'**	1.89 (1.67 – 2.13)
**Year''**	0.42 (0.32 – 0.56)
**Year'''**	8.96 (3.40 – 23.61)
Individual Characteristics	
**Previous births**	1.30 (1.30 – 1.31)
**Payment**	
Private Insurance	Reference
Medicaid	3.69 (3.62 – 3.77)
Self-Pay	2.29 (2.19 – 2.39)
Other	3.35 (3.23 – 3.48)
**Marital Status**	
Married	Reference
Unmarried	2.94 (2.89 – 2.98)
County Characteristics	
**Rurality**	
Urban	Reference
Rural Adjacent	0.82 (0.74 – 0.92)
Rural Remote	0.92 (0.81 – 1.05)
**Employed per 1,000**	1.00 (1.00 – 1.00)
**Obstetricians per 1,000**	0.92 (0.57 – 1.46)
Interactions	
**Rurality*Year'**	
Rural Adjacent	1.01 (0.97 – 1.05)
Rural Remote	0.97 (0.93 – 1.01)
**Rurality*Year''**	
Rural Adjacent	0.98 (0.90 – 1.07)
Rural Remote	1.02 (0.92 – 1.12)
**Rurality*Year'''**	
Rural Adjacent	1.07 (0.78 – 1.47)
Rural Remote	0.96 (0.67 – 1.37)
**Employed per 1,000*Year'**	1.00 (1.00 – 1.00)
**Employed per 1,000*Year''**	1.00 (1.00 – 1.00)
**Employed per 1,000*Year'''**	0.99 (0.99 – 1.00)
**Obstetricians per 1,000*Year'**	1.07 (0.92 – 1.23)
**Obstetricians per 1,000*Year''**	0.83 (0.61 – 1.14)
**Obstetricians per 1,000*Year'''**	1.44 (0.47 – 4.44)

© 2021 Patrick SW et al. *JAMA Health Forum.*

eTable 2. Predicted Probability of Hepatitis C Infection among Non-Hispanic White and American Indian or Alaskan Native People without a 4-Year Degree by County Characteristics, 2009-2019.

		**2009 % (95%CI)**	**2010 % (95%CI)**	**2011 % (95%CI)**	**2012 % (95%CI)**	**2013 % (95%CI)**	**2014 % (95%CI)**	**2015 % (95%CI)**	**2016 % (95%CI)**	**2017 % (95%CI)**	**2018 % (95%CI)**	**2019 % (95%CI)**
**Rurality**	Rural Remote	0.24% (0.18%-0.31%)	0.27% (0.21%-0.32%)	0.29% (0.24%-0.34%)	0.31% (0.27%-0.36%)	0.34% (0.31%-0.38%)	0.38% (0.34%-0.41%)	0.41% (0.37%-0.45%)	0.46% (0.41%-0.50%)	0.50% (0.43%-0.57%)	0.56% (0.46%-0.65%)	0.62% (0.50%-0.75%)
	Rural Adjacent	0.20% (0.16%-0.25%)	0.23% (0.19%-0.27%)	0.26% (0.22%-0.30%)	0.29% (0.26%-0.32%)	0.33% (0.30%-0.36%)	0.38% (0.35%-0.40%)	0.43% (0.40%-0.46%)	0.49% (0.44%-0.54%)	0.56% (0.50%-0.63%)	0.65% (0.55%-0.75%)	0.75% (0.61%-0.88%)
	Urban	0.25% (0.23%-0.28%)	0.28% (0.26%-0.31%)	0.32% (0.29%-0.34%)	0.35% (0.33%-0.38%)	0.40% (0.37%-0.43%)	0.45% (0.42%-0.48%)	0.51% (0.48%-0.54%)	0.58% (0.54%-0.62%)	0.66% (0.61%-0.71%)	0.75% (0.69%-0.82%)	0.86% (0.78%-0.95%)
**Employment Rate**	10th Percentile	0.16% (0.14%-0.18%)	0.19% (0.17%-0.21%)	0.24% (0.21%-0.26%)	0.30% (0.27%-0.32%)	0.37% (0.35%-0.40%)	0.47% (0.43%-0.51%)	0.59% (0.54%-0.64%)	0.73% (0.66%-0.80%)	0.90% (0.80%-1.01%)	1.11% (0.97%-1.26%)	1.37% (1.16%-1.58%)
	90th Percentile	0.36% (0.30%-0.43%)	0.33% (0.29%-0.37%)	0.35% (0.32%-0.39%)	0.37% (0.34%-0.41%)	0.39% (0.36%-0.42%)	0.41% (0.38%-0.44%)	0.43% (0.40%-0.46%)	0.44% (0.41%-0.48%)	0.46% (0.42%-0.50%)	0.47% (0.42%-0.52%)	0.48% (0.42%-0.54%)
**Obstetrician Rate**	10th Percentile	0.26% (0.22%-0.31%)	0.29% (0.25%-0.33%)	0.32% (0.29%-0.36%)	0.36% (0.32%-0.39%)	0.40% (0.37%-0.43%)	0.44% (0.41%-0.47%)	0.50% (0.46%-0.53%)	0.56% (0.52%-0.60%)	0.63% (0.57%-0.68%)	0.71% (0.64%-0.78%)	0.80% (0.71%-0.90%)
	90th Percentile	0.25% (0.22%-0.27%)	0.27% (0.25%-0.30%)	0.30% (0.28%-0.33%)	0.34% (0.32%-0.37%)	0.38% (0.36%-0.41%)	0.43% (0.41%-0.46%)	0.49% (0.46%-0.52%)	0.56% (0.52%-0.59%)	0.63% (0.59%-0.68%)	0.72% (0.67%-0.78%)	0.83% (0.75%-0.90%)

*After accounting for year, previous births, payment source, marital status, interactions between county characteristics and year.

© 2021 Patrick SW et al. *JAMA Health Forum.*

eFigure 2. County Geographic Variation in Hepatitis C Infections Among Pregnant People, US 2009.

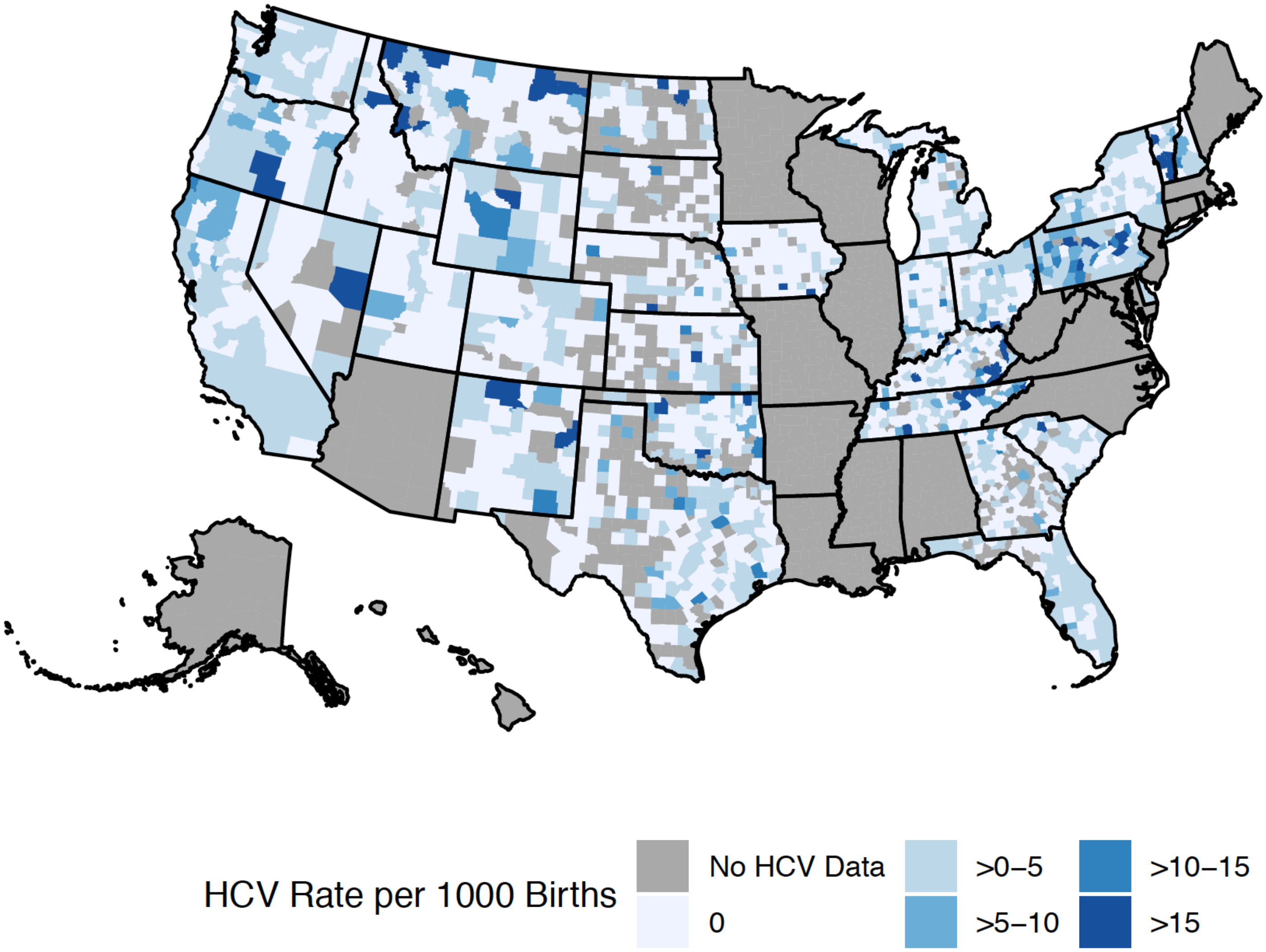

© 2021 Patrick SW et al. *JAMA Health Forum.*

eFigure 3. County Geographic Variation in Hepatitis C Infections Among Pregnant People, US 2010.

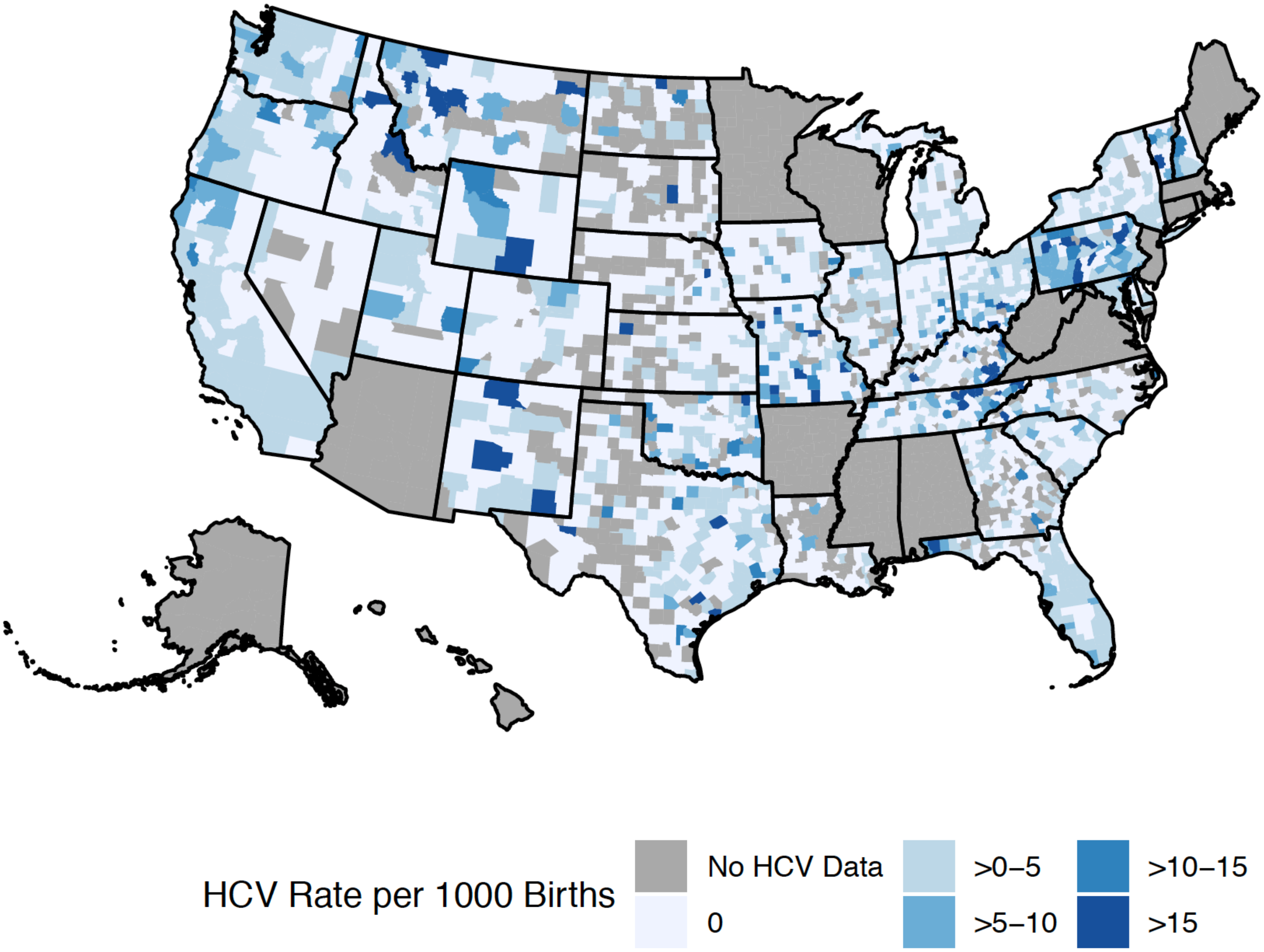

© 2021 Patrick SW et al. *JAMA Health Forum.*

eFigure 4. County Geographic Variation in Hepatitis C Infections Among Pregnant People, US 2011.

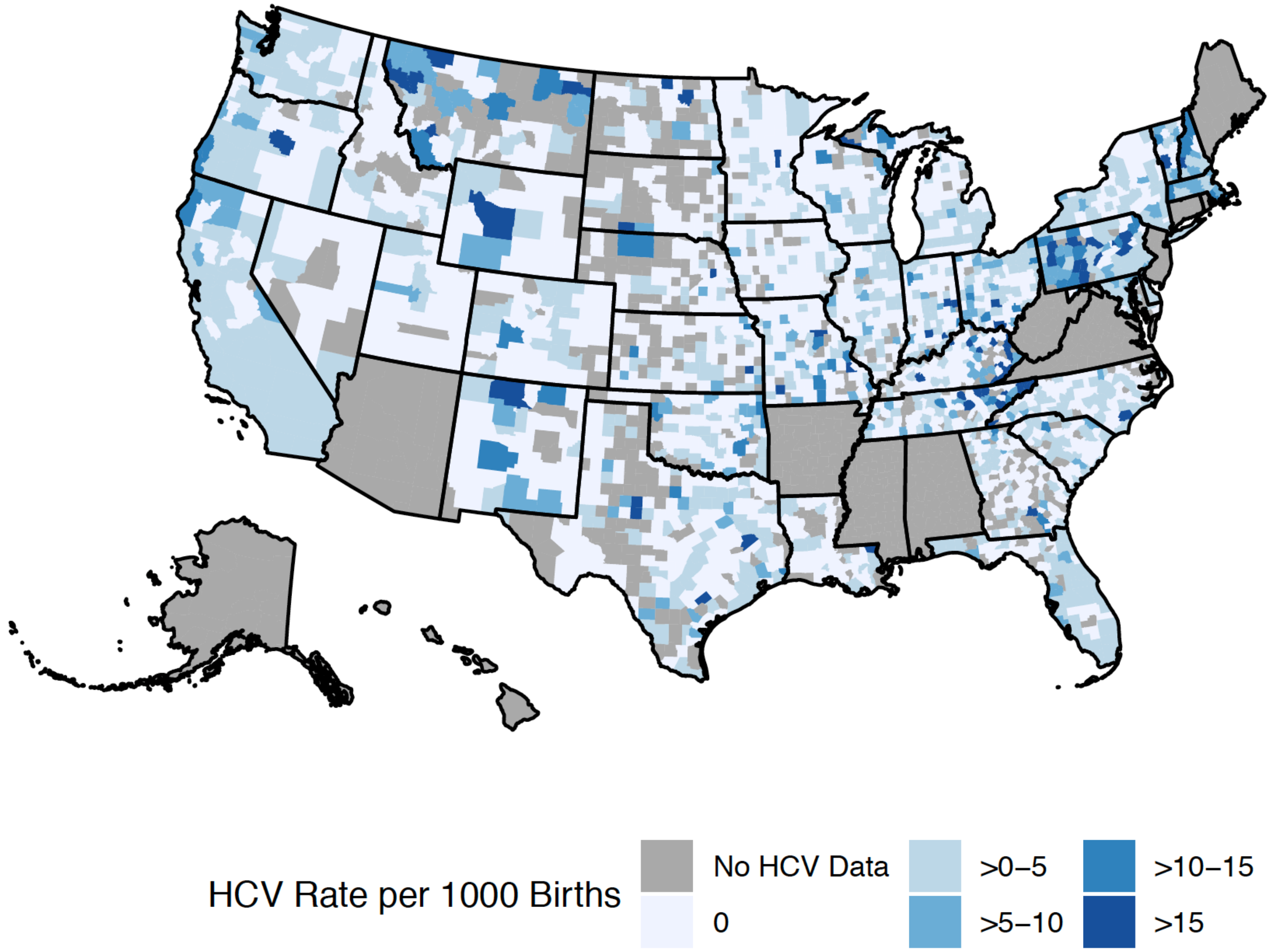

© 2021 Patrick SW et al. *JAMA Health Forum.*

eFigure 5. County Geographic Variation in Hepatitis C Infections Among Pregnant People, US 2012.

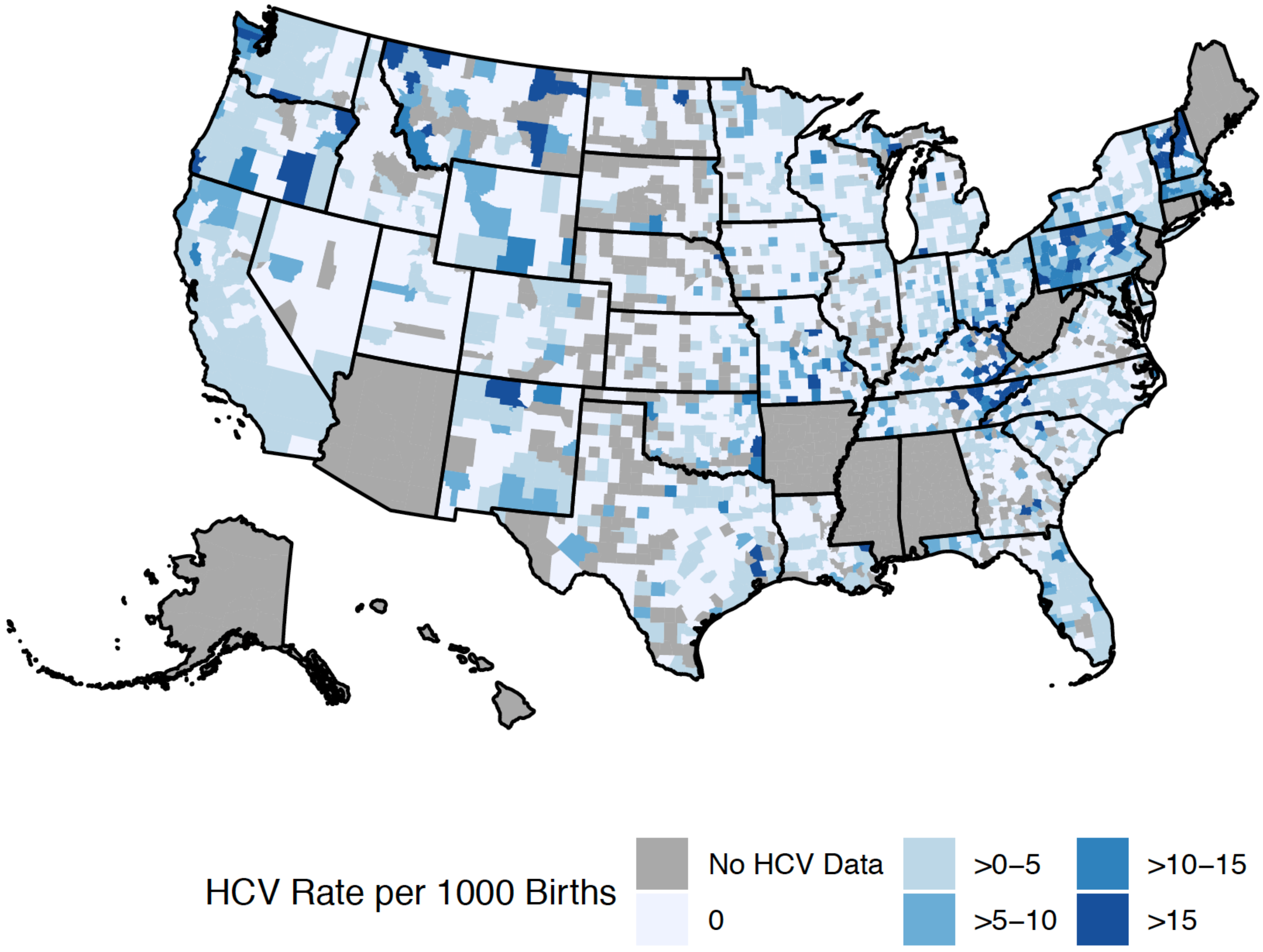

© 2021 Patrick SW et al. *JAMA Health Forum.*

eFigure 6. County Geographic Variation in Hepatitis C Infections Among Pregnant People, US 2013.

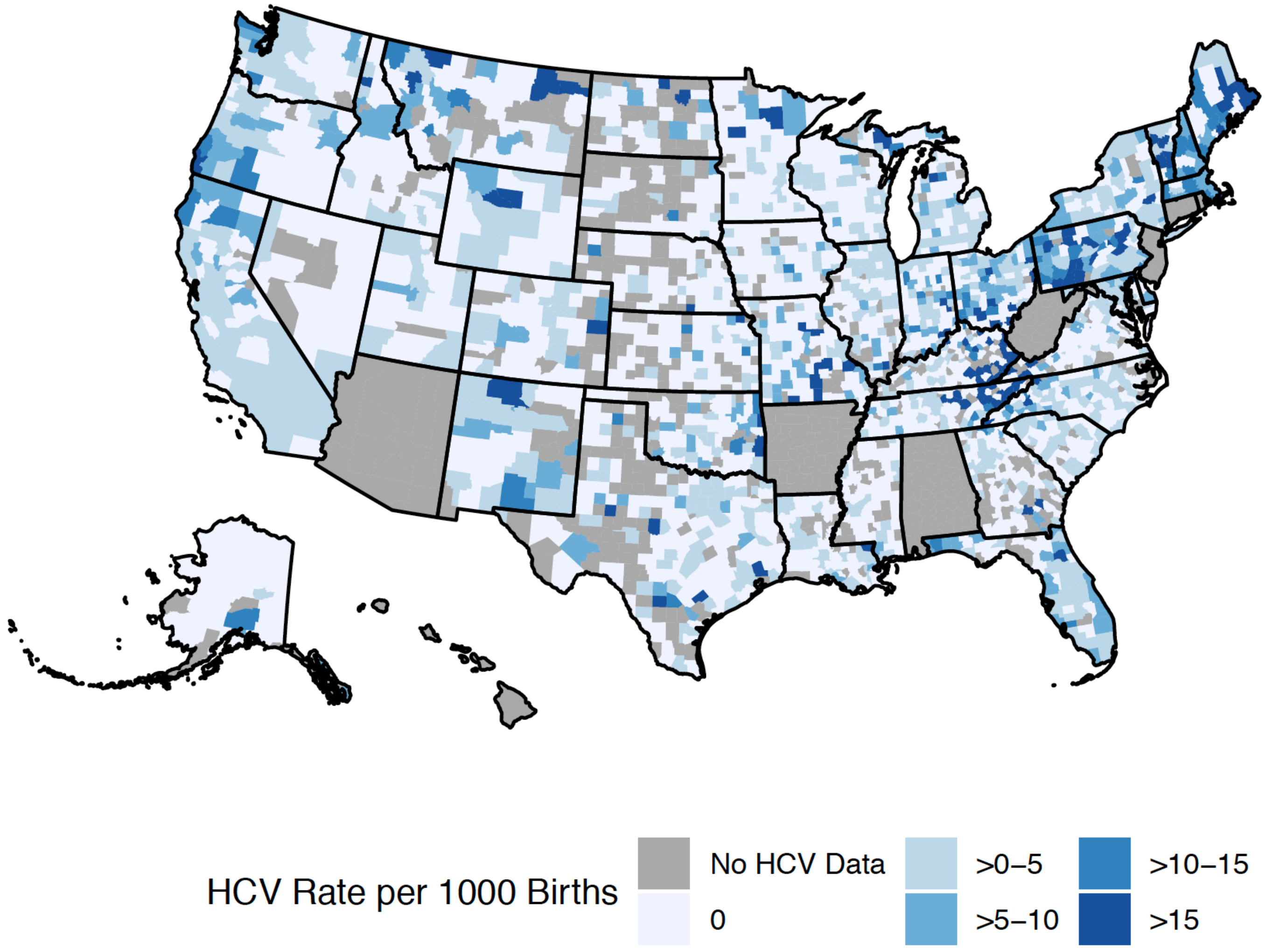

© 2021 Patrick SW et al. *JAMA Health Forum.*

eFigure 7. County Geographic Variation in Hepatitis C Infections Among Pregnant People, US 2014.

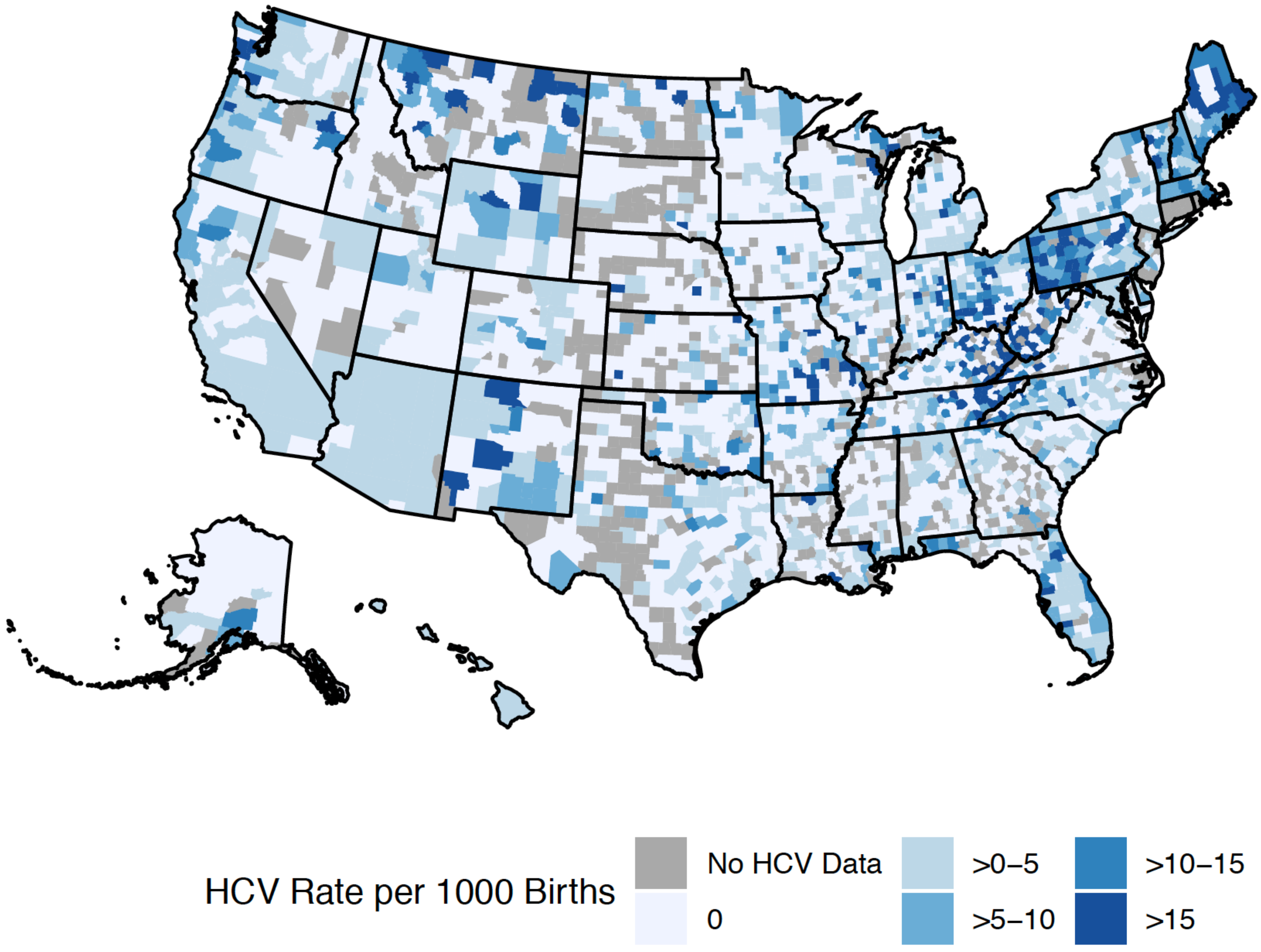

© 2021 Patrick SW et al. *JAMA Health Forum.*

eFigure 8. County Geographic Variation in Hepatitis C Infections Among Pregnant People, US 2015.

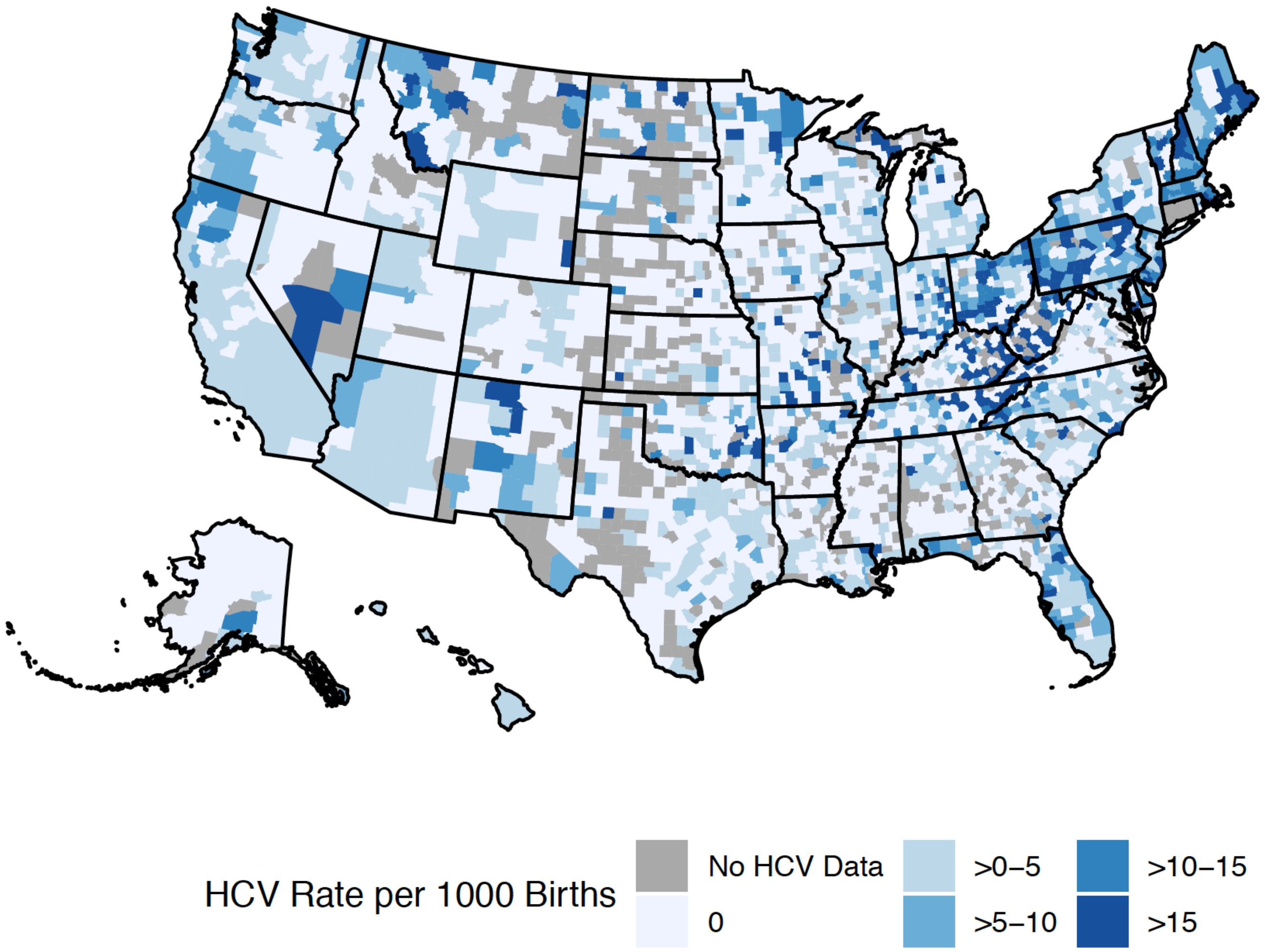

© 2021 Patrick SW et al. *JAMA Health Forum.*

eFigure 9. County Geographic Variation in Hepatitis C Infections Among Pregnant People, US 2016.

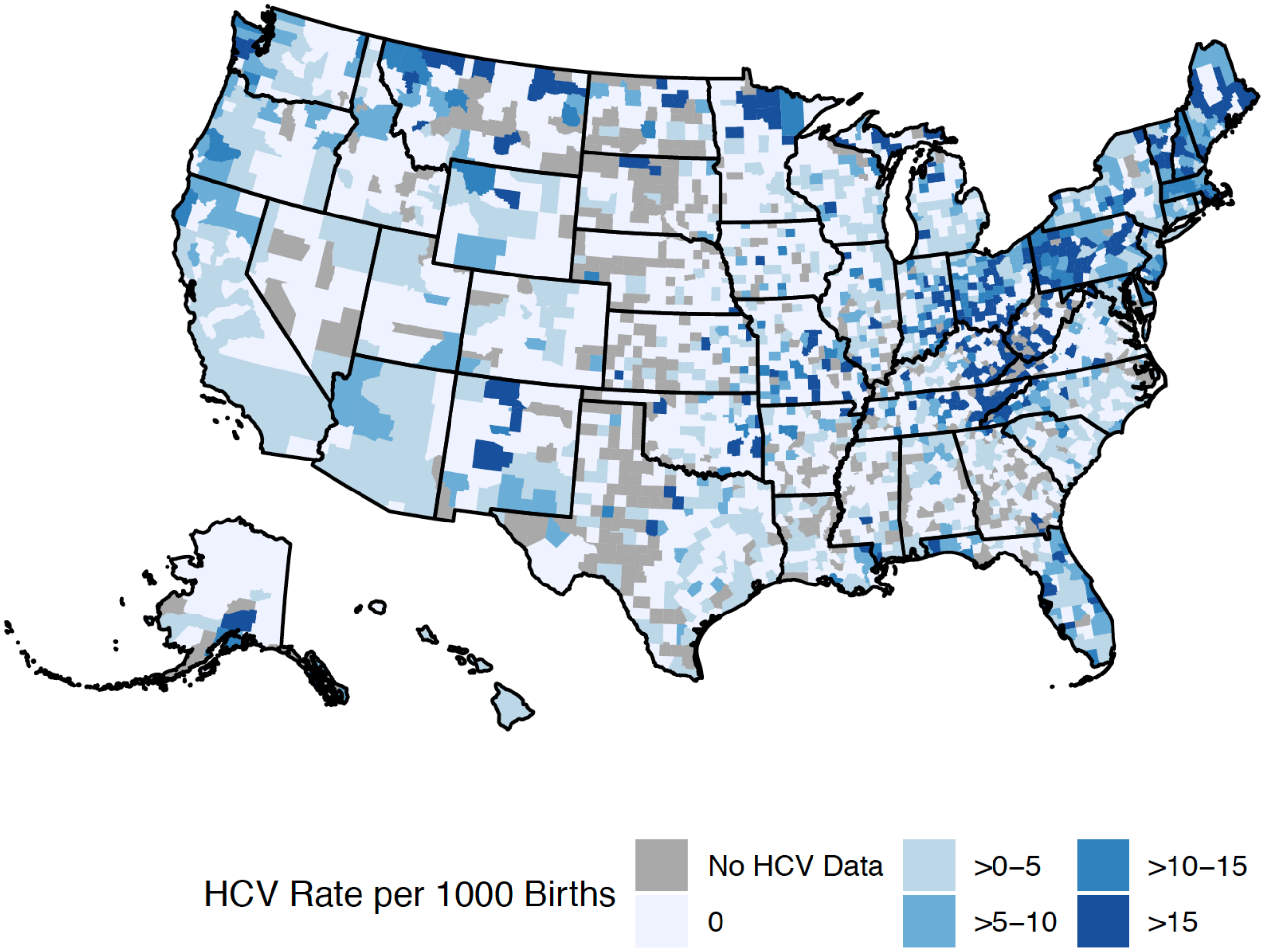

© 2021 Patrick SW et al. *JAMA Health Forum.*

eFigure 10. County Geographic Variation in Hepatitis C Infections Among Pregnant People, US 2017.

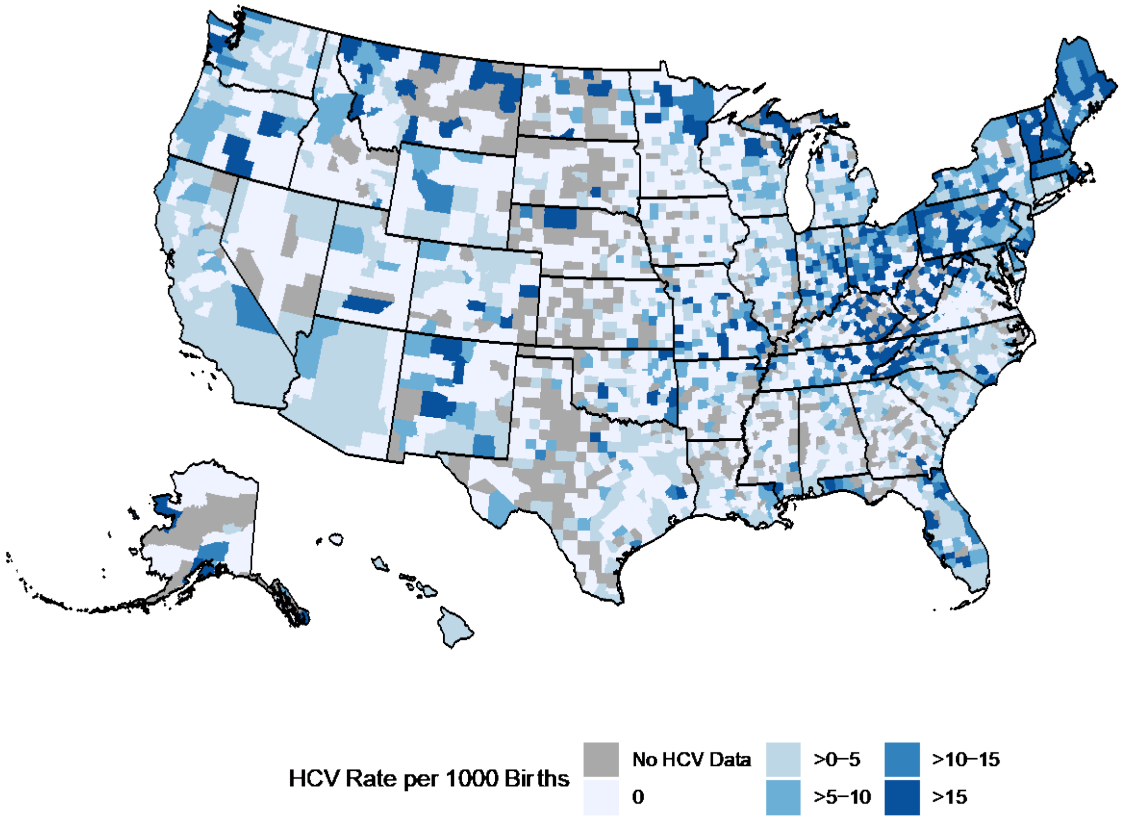

© 2021 Patrick SW et al. *JAMA Health Forum.*

eFigure 11. County Geographic Variation in Hepatitis C Infections Among Pregnant People, US 2018.

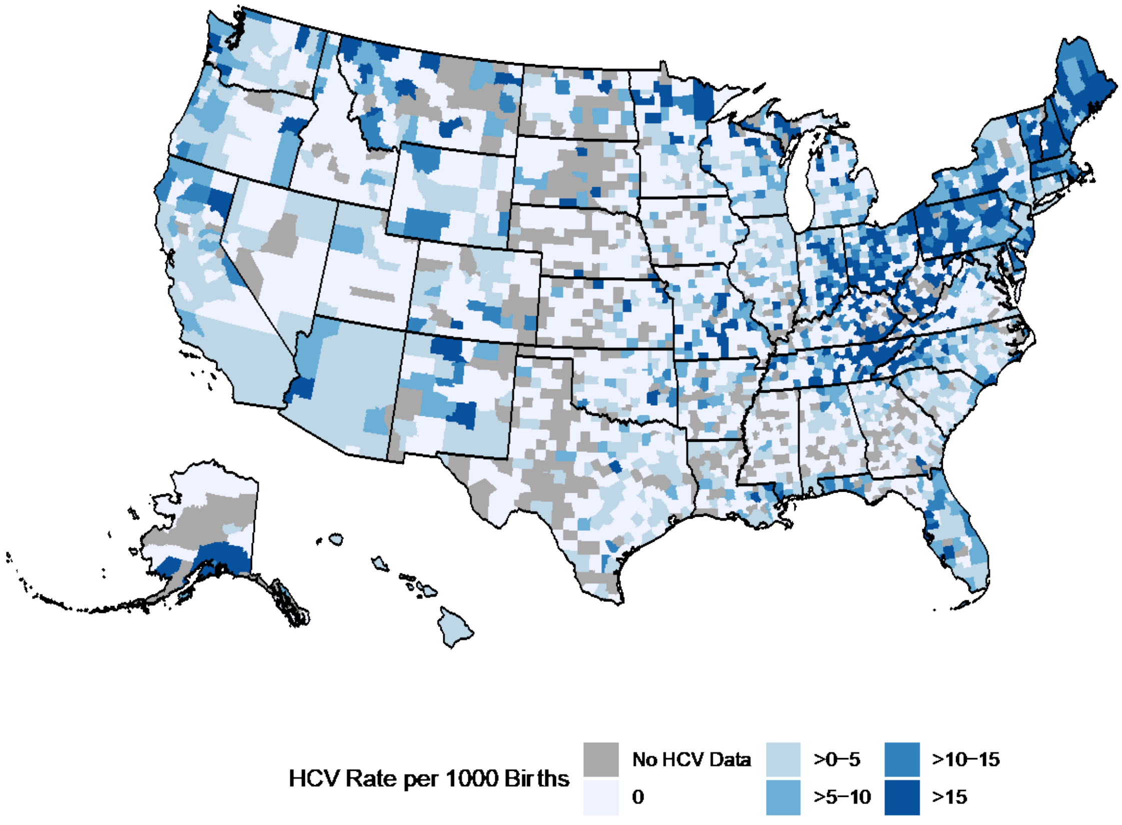

© 2021 Patrick SW et al. *JAMA Health Forum.*
